# Herpes zoster and human papillomavirus vaccination opportunities identified using electronic prompts at the time of scheduling influenza or COVID-19 vaccines

**DOI:** 10.1177/17151635231188343

**Published:** 2023-07-30

**Authors:** Sherilyn K.D. Houle, Mhd. Wasem Alsabbagh, Nancy M. Waite

**Affiliations:** School of Pharmacy, University of Waterloo, Kitchener, Ontario; School of Pharmacy, University of Waterloo, Kitchener, Ontario; School of Pharmacy, University of Waterloo, Kitchener, Ontario

## Abstract

**Background:**

Due to workload and competing priorities, vaccination-related interactions in community pharmacies tend to be more reactive than proactive. The aim of this study is to determine the proportion of users of a web-based scheduling system for influenza and COVID-19 vaccines who may be eligible for herpes zoster or human papillomavirus (HPV) vaccination and interested in discussing these vaccines with a pharmacist.

**Methods:**

Individuals scheduling an influenza or COVID-19 vaccine at a pharmacy using the MedEssist platform between October 2021 and March 2022 were asked about their vaccination status against HPV (if aged 9-45) or herpes zoster (if aged ≥50). Those who were unvaccinated or unsure were asked to indicate their willingness to discuss this with a pharmacist. Logistic regression was performed to identify patient characteristics associated with responses to these screening questions.

**Results:**

Among 36,659 bookings by those aged 9 to 45 and 55,728 by those aged ≥50 that included responses to screening questions, 70.1% and 55.5% were potentially unvaccinated against HPV and herpes zoster, respectively, with approximately 1 in 5 also indicating willingness to have a discussion with the pharmacist. Those scheduling appointments for COVID-19 vaccines were significantly less likely to be vaccinated against HPV or herpes zoster and less willing to discuss this with a pharmacist than those seeking influenza vaccination.

**Discussion:**

Automated prompts while booking influenza or COVID-19 vaccinations have the potential to identify vaccine-willing individuals who may benefit from further discussion on their vaccination needs.

**Conclusion:**

Community pharmacies can leverage available technology to support the efficient and effective identification of individuals eligible for vaccination.

**Knowledge into Practice**

- Community pharmacy vaccination services have the potential to improve population vaccination rates; however, efficiently identifying under- or unimmunized individuals and initiating discussion within existing workload can be a challenge.
- This study presents the case-finding potential of using automated HPV and herpes zoster vaccine screening questions at the time of booking an appointment for influenza or COVID-19 vaccination online.
- Leveraging online booking technology to identify patients who may benefit from an assessment of other vaccination needs offers an efficient strategy to initiate vaccine-related conversations in pharmacy practice.
- Future research will examine the effectiveness of these screening efforts at generating conversations between patients and pharmacists and subsequent receipt of indicated vaccines.

**Mise En Pratique Des Connaissances**

- Les services de vaccination des pharmacies communautaires ont le potentiel d’améliorer les taux de vaccination de la population; cependant, il peut s’avérer difficile d’identifier efficacement les personnes sous-vaccinées ou non vaccinées et d’entamer une discussion en plus de la charge de travail actuelle.
- Cette étude présente le potentiel de détection de cas par l’utilisation de questions automatisées de dépistage des vaccins contre le VIH et l’herpès zoster au moment de la prise de rendez-vous en ligne pour la vaccination contre la grippe ou la COVID-19.
- L’utilisation de la technologie de réservation en ligne pour l’identification des patients qui pourraient bénéficier d’une évaluation de leurs besoins en matière de vaccination constitue une stratégie efficace pour entamer des discussions sur les vaccins dans le cadre de la pratique pharmaceutique.
- Les recherches futures examineront l’efficacité de ces efforts de dépistage pour susciter des conversations entre les patients et les pharmaciens, et la réception ultérieure de vaccins indiqués.

## Introduction

Vaccination rates among Canadian adults are suboptimal across most vaccine-preventable diseases, such as influenza, pneumococcal disease, tetanus and pertussis.^1^ For human papillomavirus (HPV), a systematic review and meta-analysis of Canadian data estimated an HPV vaccination uptake among eligible people of 55.9% overall, ranging from 66.9% among those aged ≤18 years to 13.6% in those aged >18 years.^2^ Additionally, as of February 2021, only 27% of Canadian adults aged ≥50 years self-reported being vaccinated against herpes zoster.^3^ With contributing factors such as shifts to virtual medical visits, the pausing of school-based vaccination programs and a focus on COVID-19 vaccination efforts during the pandemic, many vaccine-eligible Canadians remain unprotected against herpes zoster and HPV.^4,5^ Current Canadian guidelines recommend HPV vaccination for all individuals aged ≥9 to <27 years (with vaccination of those ≥27 years recommended if ongoing risk of exposure and use in those aged >45 years being off-label)^6-8^ and herpes zoster vaccination for all adults aged ≥50 without contraindications and adults aged 18 to 49 who are immunocompromised.^9^

Pharmacies are a highly trusted and convenient setting for the administration of vaccines and in 2019/2020 became the most common place for influenza vaccination among Canadians.^10^ Pharmacists are well positioned and capable of having vaccine conversations with patients, including those expressing vaccine hesitancy; however, due to workload and competing demands, such interactions have been found to be more reactive than proactive.^11^ As a result, patients who approach the pharmacist with a question or a desire to be vaccinated are likely to have their needs met, but those who do not know they are eligible for vaccination may remain unaware. Research on influenza vaccine uptake in Ontario has identified that pharmacies reach a broad population demographic,^12^ including younger adults who value the convenience of accessing vaccination services outside of typical medical clinic hours and potentially without requiring an appointment.^13^

Technology now allows patients to schedule vaccine appointments with pharmacies online—a service that was especially valuable during COVID-19 mass vaccination efforts and continues to be used to book influenza and other vaccinations. In August 2020, MedEssist launched its vaccination service platform, which is currently in use across Canada. At present, approximately 300 independent community pharmacies use the platform, with 83% located in Ontario. Recognizing an opportunity to identify other potentially indicated vaccines among those scheduling a COVID-19 or influenza vaccine, MedEssist integrated additional vaccine screening questions into the platform in 2021, specifically targeting herpes zoster and HPV vaccines. However, the effectiveness of these questions at identifying individuals who may be in need of vaccination is unknown. It is also unknown whether the type of vaccine being scheduled (influenza vs COVID-19) is associated with differences in vaccination status with other vaccines or willingness to discuss other vaccination needs with a pharmacist.

The objective of this work is to determine the proportion of individuals using the scheduling system for influenza or COVID-19 vaccines who may also be eligible for and interested in discussing vaccination against herpes zoster or HPV using these automated prompts, including their demographic characteristics (age, sex). Additionally, given significant differences in vaccination rates for the primary series of COVID-19 vaccine versus influenza vaccine among Canadians (80.5% vs 39%, respectively),^14,15^ this work will also compare results across bookings for each vaccine type with the hypothesis that differences in overall willingness and motivation to be vaccinated with pandemic versus routine vaccines may differ across these groups. Equipped with this knowledge, pharmacies can consider the opportunities available to them to improve vaccination rates and tailor their case finding efforts to individuals who are more likely to have an unmet vaccination need and be receptive to an active offer of vaccination by a pharmacist.

## Methods

Individuals using the MedEssist program to schedule an influenza or COVID-19 vaccine for a person aged ≥9 years through a participating community pharmacy (approximately 300 locations) between October 2021 and March 2022 were included in the analysis. Based on the age input during the booking process and consistent with Canadian-labelled indications, the system asked those aged 9 to 45 years about their vaccination status against HPV and those aged ≥50 years about their vaccination status against herpes zoster. Those who responded that they were unvaccinated or unsure were further asked about their interest in speaking with a pharmacist about their vaccination. Answering these questions was optional. Data on the respondent’s self-reported sex were also collected.

SAS® 9.4 was used for data analysis. Age of respondents was categorized as 9 to 17, 18 to 24, 25 to 29, 30 to 39, 40 to 49, 50 to 59, 60 to 69, or ≥70 years. Respondents were stratified depending on the vaccine they were booking for (either COVID-19 or influenza vaccine). Respondents who answered, “I do not know” to having received HPV or herpes zoster vaccine were considered as “no.” Similarly, respondents who did not answer the question pertaining to being interested in speaking with a pharmacist about HPV/herpes zoster vaccine were considered as “no.” First, we described the age and sex distribution of respondents who answered the additional screening questions for HPV or herpes zoster vaccines, those who were unvaccinated against them (or with unknown status) and those who were interested in speaking with the pharmacist about them. Then, we fitted a logistic regression to examine the association between the vaccine the respondent was booking for and being vaccinated against HPV (for respondents aged 9-45) and against herpes zoster (for respondents aged ≥50). Last, for unvaccinated respondents, we fitted a logistic regression between the vaccine the respondent was booking for and being interested in speaking with the pharmacist about the vaccine. In both models, we adjusted for the age and sex of the respondent. The odds ratio (OR) with corresponding 95% confidence interval (95% CI) for both the crude and adjusted model was calculated.

A data-sharing agreement, including processes for ensuring anonymity of users and their responses to the screening questions, was established between the University of Waterloo and MedEssist. Ethics approval for the study was received from the University of Waterloo Office of Research Ethics (ORE 44149).

## Results

Over the study period, 152,951 bookings for an influenza or COVID-19 vaccine appointment were made using the platform, with 36,659 and 55,728 individuals answering questions about their vaccine status against HPV or herpes zoster, respectively. Among those, 26,015 (70.1%) were unvaccinated or had uncertain vaccination status against HPV and 30,937 (55.5%) against herpes zoster, with 4306 (16.6%) and 6537 (21.1%), respectively, expressing interest in discussing their vaccination needs with a pharmacist. Responses by vaccine type and demographic factors of respondents are presented in Tables 1 and 2.

**Table 1:** Screening question responses by age and sex among those eligible for the HPV vaccine

	Booking Influenza Vaccine			Booking COVID-19 Vaccine		
Age Range	Female	Male	Nonbinary, other, prefer to self-describe	Female	Male	Nonbinary, other, prefer to self-describe
	Individuals booking influenza or COVID-19 vaccine who answered additional screening questions, *n*					
9-17	1083	1137	7	2096	2300	29
18-24	626	419	12	2408	2317	51
25-29	355	220	1	2176	1973	37
30-39	1163	851	6	5194	4567	37
40-45	1007	707	0	3049	2814	17
	Individuals who responded that they were unvaccinated or had unknown vaccination status against HPV, *n* (% of total above)					
9-17	666 (61.5)	778 (68.4)	4 (57.1)	1497 (71.4)	1717 (74.7%)	17 (58.6)
18-24	192 (30.7)	249 (59.4)	5 (41.7)	1095 (45.5%	1580 (68.2)	22 (43.1)
25-29	159 (44.8)	165 (75)	1 (100)	1225 (56.3)	1440 (73.0)	23 (62.2)
30-39	804 (69.1)	707 (83.1)	4 (66.7)	3780 (72.8)	3644 (79.8)	29 (78.4)
40-45	843 (83.7)	626 (88.5)	0	2414 (79.2)	2315 (82.3)	14 (82.4)
	Individuals who responded that they were interested in speaking with a pharmacist about HPV vaccine, *n* (% of those unvaccinated or with unknown vaccination status)					
9-17	110 (16.5)	121 (15.6)	3 (75)	226 (15.1)	260 (15.1)	3 (17.6)
18-24	48 (25.0)	61 (24.5)	4 (80)	203 (18.5)	249 (15.8)	9 (41.0)
25-29	47 (29.6)	39 (23.6)	0	240 (19.6)	208 (14.4)	5 (21.7)
30-39	168 (21.0)	116 (16.4)	1 (25)	613 (16.2)	550 (15.1)	8 (27.6)
40-45	176 (20.9)	107 (17.1)	0	400 (16.6)	331 (14.3)	0

**Table 2:** Screening question responses by age and sex among those eligible for the herpes zoster vaccine

	Booking Influenza Vaccine			Booking COVID-19 Vaccine		
Age Range	Female	Male	Nonbinary, other, prefer to self-describe	Female	Male	Nonbinary, other, prefer to self-describe
	Individuals booking influenza or COVID-19 vaccine who answered additional screening questions, *n*					
50-59	2421	1663	3	6596	6157	23
60-69	3993	3056	1	7436	6633	14
≥70	3558	3225	6	5818	5114	11
	Individuals who responded that they were unvaccinated or had unknown vaccination status against herpes zoster, *n* (% of total above)					
50-59	1630 (67.3)	1157 (69.6)	3 (100)	4863 (73.7)	4730 (76.8)	19 (82.6)
60-69	1831 (45.9)	1570 (51.2)	1 (100)	3898 (52.4)	3761 (56.7)	6 (42.9)
≥70	1515 (42.6)	1388 (43.0)	2 (33.3)	2361 (40.6)	2199 (43.0)	3 (27.3)
	Individuals who responded that they were interested in speaking with a pharmacist about herpes zoster vaccine, *n* (% of those unvaccinated or with unknown vaccination status)					
50-59	541 (33.2)	330 (28.5)	0	994 (20.4)	893 (18.9)	4 (21.1)
60-69	487 (26.6)	456 (29.0)	0	739 (19.0)	775 (20.6)	1 (16.7)
≥70	323 (21.3)	295 (21.3)	1 (50)	365 (15.5)	333 (15.1%	0

Booking for COVID-19 vaccine was associated with lower odds of being vaccinated with HPV vaccine (crude OR 0.63, 95% CI 0.63-0.71; adjusted OR 0.62, 95% CI 0.58-0.66) and with herpes zoster vaccine (crude OR 0.61, 95% CI 0.59-0.64; adjusted OR 0.70, 95% CI 0.67-0.73) versus bookings for influenza vaccine. Similarly, booking for COVID-19 vaccine was associated with lower odds of being interested in speaking with the pharmacist about the HPV vaccine (crude OR 0.79, 95% CI 0.73-0.85; adjusted OR 0.77, 95% CI 0.71-0.84) and herpes zoster vaccine (crude OR 0.63, 95% CI 0.59-0.66; adjusted OR 0.60, 95% CI 0.57-0.64) versus booking for influenza vaccine among unvaccinated respondents. Odds ratio estimations from the adjusted logistic regression models are available in Tables 3 and 4.

**Table 3:** Odds ratio estimates of having received HPV and herpes zoster vaccines among age-eligible respondents

Characteristic	Odds Ratio Estimate	95% Wald Confidence Limits	
**HPV Vaccine**			
Booking for COVID-19 vaccine vs booking for influenza vaccine	0.621	0.580	0.664
Age			
9-17 vs 40-49	3.023	2.711	3.371
18-24 vs 40-49	7.714	6.939	8.575
25-29 vs 40-49	4.843	4.329	5.418
30-39 vs 40-49	2.041	1.841	2.264
Sex			
Female vs male	2.583	2.431	2.745
Nonbinary vs male	3.450	2.048	5.812
Other orientation vs male	2.046	0.976	4.287
Prefer not to say vs male	2.341	1.393	3.936
**Herpes Zoster Vaccine**			
Booking for COVID-19 vaccine vs booking for influenza vaccine	0.699	0.672	0.728
Age			
50-59 vs ≥70	0.247	0.235	0.261
60-69 vs ≥70	0.733	0.702	0.766
Sex			
Female vs male	1.231	1.185	1.279
Nonbinary vs male	1.397	0.376	5.189
Other orientation vs male	0.661	0.161	2.714
Prefer not to say vs male	1.439	0.672	3.079

**Table 4:** Odds ratio estimates of being interested in speaking with a pharmacist about HPV or herpes zoster vaccines among age-eligible respondents who are unvaccinated or have unknown vaccination status

Characteristic	Odds ratio estimate	95% Wald confidence limits	
**HPV Vaccine**			
Booking for COVID-19 vaccine vs booking for influenza vaccine	0.773	0.714	0.837
Age			
9-17 vs 40-49	0.926	0.835	1.028
18-24 vs 40-49	1.191	1.063	1.334
25-29 vs 40-49	1.167	1.039	1.310
30-39 vs 40-49	1.013	0.928	1.106
Sex			
Female vs male	1.173	1.098	1.253
Nonbinary vs male	1.865	0.909	3.823
Other orientation vs male	6.278	2.633	14.967
Prefer not to say vs male	1.315	0.680	2.542
**Herpes Zoster Vaccine**			
Booking for COVID-19 vaccine vs booking for influenza vaccine	0.600	0.566	0.636
Age			
50-59 vs ≥70	1.454	1.350	1.566
60-69 vs ≥70	1.389	1.289	1.498
Sex			
Female vs male	1.019	0.964	1.076
Non-binary vs male	2.152	0.393	11.791
Other orientation vs male	1.145	0.220	5.948
Prefer not to say vs male	0.438	0.102	1.881

Lower respondent age was associated with an increased likelihood of having received HPV vaccination, whereas older age was associated with having received herpes zoster vaccination, possibly reflecting age-based perceptions of risk. Lower age was associated with a higher willingness to speak with a pharmacist about herpes zoster vaccination. Regarding sex associations, individuals identifying as nonbinary or a gender orientation other than male or female had a higher likelihood of willingness to speak with a pharmacist about HPV vaccination versus those identifying as male (OR 1.865 and OR 6.278, respectively, which was statistically significant for other gender orientation), although the small sample sizes of these groups contributed to wide confidence intervals. A similar trend was observed for herpes zoster vaccination, although neither group had a statistically significant difference versus males.

## Discussion

Case finding using a vaccine scheduling system identified a high proportion of age-eligible individuals who were unvaccinated or had uncertain vaccination status against HPV (70%) and herpes zoster (55%). However, only 1 in 5 individuals reported being both potentially unprotected and interested in an assessment of their vaccination needs with a pharmacist. Booking for influenza vaccine was associated with higher odds of being vaccinated against HPV or herpes zoster and, if not vaccinated, with being interested in talking with their pharmacist about receiving the vaccine. The use of such screening questions can easily identify opportunities to improve population vaccination rates through community pharmacies. Conversations on other vaccination needs can take place either at the time of the vaccination that was initially scheduled online, during a regular medication review or at a separate dedicated appointment time.

Among our sample, the herpes zoster vaccination rate of 44.5% is notably higher than the 27% identified through previous research,^3^ suggesting that those who are proactive in scheduling influenza or COVID-19 vaccines may be more vaccine-willing overall and more likely to be vaccinated against other diseases. Despite this, a significant proportion of individuals remain unprotected even among those presenting to the pharmacy for a vaccination service, which may indicate a lack of awareness of other indicated vaccines. The unique circumstances surrounding COVID-19 vaccination should also be considered, such as vaccination requirements to be able to travel or participate in other activities during the pandemic. These factors may have encouraged individuals to be vaccinated against COVID-19 who are otherwise less interested in vaccination or are vaccine hesitant. Although not the only contributing factor, an interrupted time series analysis identified significant increases in vaccine uptake rates across most Canadian provinces in the weeks following COVID-19 vaccine mandate announcements,^16^ suggesting that such mandates may have influenced some people to be vaccinated who may not have otherwise chosen to be. This hypothesis is congruent with our finding that the proportions of individuals who were unvaccinated or had unknown vaccination status against both HPV and herpes zoster were higher among those booking a COVID-19 vaccine than among those booking an influenza vaccine; correspondingly, interest in speaking with a pharmacist about vaccination was lower. The generalizability of our findings to future vaccine seasons, including the impact and longevity of pandemic-related vaccine fatigue (defined as inertia or inaction toward vaccine information or instruction due to perceived burden and burnout),^17^ may be affected.

Case finding has been defined as a process that uses “demographics, risk factors and/or symptoms at an individual level to decide whether to apply a test or proceed with additional testing.”^18^ Although case finding has been applied to identifying individuals who may be at risk of chronic disease or vaccine-preventable disease (e.g., through running reports from dispensary software programs to identify individuals of a certain age and/or using specific medications), these efforts are often labour-intensive, as they require actively reaching out individually to those with risk factors to offer the clinical service. Additionally, both pharmacy staff and patients may be less comfortable with “cold calling” to offer a pharmacy service to potentially eligible individuals. Automation of this process to identify individuals who are at risk of vaccine-preventable illness and who already have interest in the clinical service may be more efficient and easier to manage within existing community pharmacy workloads. To our knowledge, this is the first study to report on the potential case-finding opportunities that can be realized through automated screening questions built into a vaccine scheduling program.

Although effective at identifying individuals who may benefit from an assessment of their vaccination needs, this method unfortunately found low expression of interest in having a discussion with a pharmacist. Although some of this may be representative of underlying vaccine hesitancy, other factors to consider include concerns about the time required to have such a conversation and the need to obtain vaccination records, questions about the motivation behind offering vaccination and any associated out-of-pocket costs or uncertainty about a pharmacist’s clinical knowledge of vaccines. Strategies to increase this willingness may include offering access to resources from reputable organizations on the risk of the vaccine-preventable disease, the safety and effectiveness data on available vaccines and information on eligibility criteria for publicly funded vaccines or the potential for coverage through a private insurer in follow-up correspondence before asking users to indicate willingness to have this discussion. Users can also be reassured that the collating of a complete vaccination history is not a prerequisite to having a discussion about potentially indicated vaccines. It is also possible that willingness to discuss additional vaccines with a pharmacist may differ when such a conversation is initiated in person versus through expressing interest online. We therefore recommend that pharmacy staff continue to actively engage with patients in discussions on vaccination when appropriate.

Limitations of this data include the low response rate for completing the optional screening questions (60%), uncertain accuracy of self-report of vaccination status against HPV and herpes zoster and lack of information on which herpes zoster vaccine may have been received in the past. As the National Advisory Committee on Immunization recommends that those who have previously received the live attenuated herpes zoster vaccine also receive the 2-dose series of the recombinant vaccine,^9^ respondents may have indicated that they are vaccinated even if they have only received the live attenuated herpes zoster vaccine. Similarly, respondents who initiated but did not complete the vaccine series with either the HPV or recombinant herpes zoster vaccine may similarly report being vaccinated. It must also be recognized that our sample consists entirely of individuals scheduling a vaccine appointment online, requiring Internet access and the ability to book the appointment or access to another person able to provide this for them and may not represent those without such access. Finally, it is uncertain whether vaccination rates or willingness to discuss vaccinations with a pharmacist may differ among those who opted not to answer these screening questions.

## Conclusion

The use of automated electronic prompts at the time of scheduling a vaccination appointment online may offer an effective and efficient approach to identify vaccine-willing individuals with unmet vaccination needs. Future research will explore the effectiveness of this prompt at facilitating vaccine conversations and the subsequent impact on vaccination uptake as well as the feasibility of offering appointment-based and remunerated vaccination assessments by pharmacists. In addition to the active offer of vaccination services to individuals presenting to community pharmacies, the automation of case finding efforts represents an opportunity to further support efforts to improve vaccination rates and protect our patients against the morbidity and mortality associated with vaccine-preventable disease. ■
